# Detection frequency of SARS-CoV-2 over the different waves of COVID-19 between 2020 and 2022 in Cameroon

**DOI:** 10.11604/pamj.2025.52.10.44407

**Published:** 2025-09-10

**Authors:** Pauliana Vanessa Ilouga, Jules Brice Tchatchueng-Mbougua, Ripa Mohamadou Njankouo, Loique Landry Essengue Messanga, Paul Alain Tagnouokam-Ngoupo, Serge Alain Sadeuh-Mba, Ngu Njei Abanda, Estelle Longla Madaha, Sebastian Kenmoe, Valerie Donkeng, Ariane Nzouankeu, Elodie Ngo Malabo, Constant Anatole Pieme, Tania Crucitti, Chavely Gwladys Monamele, Serge Tchatchouang, Abdou Fatawou Modiyinji, Aristide Mounchili Njifon, Moise Henri Moumbeket Yifomnjou, Delia Djuicy, Ronald Perraut, Habiba Kemkoi, Hermann Landry Njifon, Mathurin Cyrille Tejiokem, Sara Eyangoh, Richard Njouom

**Affiliations:** 1 Centre Pasteur of Cameroon, Yaoundé, Cameroon,; 2 School of Health Sciences, Catholic University of Central Africa, Yaoundé, Cameroon,; 3 Department of Biochemistry, University of Yaounde I, Yaoundé, Cameroon,; 4 Centre Pasteur of Cameroon, Garoua Annex, Garoua, Cameroon

**Keywords:** SARS-CoV-2, COVID-19, detection frequency, Yaounde, Cameroon

## Abstract

**Introduction:**

Cameroon faced several waves of COVID-19 epidemics between 2020 and 2022. The epidemic peaks were characterized by variations in the number of positive cases and major variants. The aim of this study was to determine the frequency of COVID-19 in Cameroon during the different epidemic waves.

**Methods:**

nasopharyngeal samples were collected in different regions of Cameroon between 2020 and 2022. The Daan Gene kit (DaAn Gene, Guangzhou, Guangdong Province, China) was used to detect SARS-CoV-2 in the samples by RT-PCR assay. Excel 2013 software was used to record participants´ sociodemographic characteristics (age, sex, sampling date) and SARS-CoV-2 test results. Statistical analyses were performed using IBM SPSS version 25 software.

**Results:**

from 16 March 2020 to 31 December 2022, 142,850 samples were tested. Participants ranged in age from 1 to 99 years with a M/F sex ratio of 1.32. Of the participants tested, 17,463 (12.2%) were positive for SARS-CoV-2. The SARS-CoV-2 detection rate decreased over time and was highest in 2020 (15.6%; 7255/46466) as opposed to 2021 (12.7%; 8859/69867) and 2022 (5.1%; 1349/26383). Four peaks of COVID-19 circulation were identified: May 2020, March 2021, September 2021 and December 2021. Risk factors for increased detection of SARS-CoV-2 were being older than 65 years and being from the Littoral region.

**Conclusion:**

the SARS-CoV-2 positivity rate in Cameroon decreased over the years, probably due to compliance with the barrier measures implemented by the Cameroonian government to reduce transmission rates.

## Introduction

COVID-19 (Coronavirus Disease 2019) is an infectious disease caused by severe acute respiratory syndrome coronavirus 2 (SARS-CoV-2). This new coronavirus emerged in the city of Wuhan, Hubei Province, China, in December 2019 [1] and then spread around the world, causing different waves of COVID-19 epidemics. SARS-CoV-2 is an RNA virus of the family *Coronaviridae*, subfamily *Orthocoronavirinae*, genus *Betacoronavirus* [2]. The virions are spherical with a diameter of approximately 80nm [3]. The viral genome of approximately 30Kb encodes four structural proteins, the spike, membrane, envelope and nucleocapsid proteins, which are essential for viral assembly. In addition, the viral genome encodes sixteen non-structural proteins that play a role in viral RNA synthesis and nine accessory proteins that confer a selective advantage to the infected host (Bai *et al*. 2024). These proteins also serve as targets for several diagnostic tools to screen for the presence of SARS-CoV-2.

The SARS-CoV-2 pandemic has affected all continents, resulting in an enormous number of cases, hospitalizations and deaths. Worldwide, 767,518,723 cases of COVID-19 and 6,947,192 deaths have been reported to the WHO as of 28 June 2023. In Africa, 9,540,096 cases of COVID-19 were reported between 31 December 2019 and 31 December 2022 [4]. To prevent new cases of SARS-CoV-2 infection, the World Health Organization (WHO) recommended barrier measures, including social distancing, hand washing, continuous use of masks, use of hydroalcoholic gel and vaccination [5].

In Cameroon, the data provided by the Public Health Emergency Operations Coordination Centre on 10 January 2024 for the last epidemiological week of 2023, from 25 to 31 December 2023, showed 125,248 confirmed cases of COVID-19 and 1974 recorded deaths [6]. All reference laboratories for SARS-CoV-2 diagnosis in Cameroon contributed to these statistics, including the Centre Pasteur Cameroon (CPC), which was one of the first laboratories to initiate diagnosis at the national level. The CPC received nasopharyngeal samples collected in all regions of the country. The aim of this article is to present the frequency of detection of SARS-CoV-2 in Cameroon throughout the pandemic. Socio-demographic factors associated with higher infection rates are also discussed.

## Methods

**Study design:** we conducted a transversal study to determine the detection frequency of SARS-CoV-2 during COVID-19 epidemics recorded in Cameroon between 2020 and 2022.

**Study setting:** the study was conducted at the Virology Laboratory of the Centre Pasteur du Cameroun. The COVID-19 diagnostic sampling locations around Cameroon (Centre, Littoral, West, South, North, South-West, and North-West) provided the samples that were used. Nasopharyngeal samples were stored in a Virological Transport Medium and triple-wrapped for security and sent within 24 hours to the Centre Pasteur of Cameroon for SARS-CoV-2 testing by real-time PCR assay.

**Participants:** all participants with fever and respiratory illness within the previous ten days and who presented at the state-identified hospital facilities within each region for SARS-CoV-2 testing were eligible to participate in the study.

**Variables:** the main variables in this study were the SARS-CoV-2 positivity status and the participant´s socio-demographic status including date of illness, age, gender and region of residence.

**Data sources/measurement:** sociodemographic data was collected using individual patient identification forms that accompanied each sample. Once at the CPC, the nasopharyngeal samples collected from the hospital facilities were immediately processed for RNA extraction and subsequently SARS-CoV-2 testing. Following the manufacturer's instructions, 50 µl of RNA was extracted from the nasopharyngeal swabs using the DaAn Gene nucleic acid extraction kit (DaAn Gene, Guangzhou, Guangdong Province, China). RT-PCR was then performed using the DaAn Gene Kit (DaAn Gene, Guangzhou, Guangdong Province, China) which targets the N and ORF1ab genes of SARS-CoV-2. The kit includes an internal control targeting the human RNase P gene to validate the RT-PCR result. The master mix contains 17 µl of PCR reaction mix (liquid A) and 3 µl of PCR enzyme (liquid B), which are mixed with 5 µl of the extracted sample to give a final volume of 25 µl. RT-PCR amplification was performed using QuantStudio 7 Flex (Applied Biosystems). Thermal cycling conditions for cDNA synthesis included 15 min at 50°C and 15 min at 95°C followed by 45 amplification cycles at 94°C for 15 sec and 55°C for 45 sec. The assay was validated by the amplification of the internal control RNase P. Results were considered positive if amplification of the N and ORF1ab genes had a threshold (Ct) of less than 37. Results were considered negative if amplification of the N and ORF1ab genes had a threshold (Ct) greater than 37. Results were considered indeterminate if amplification of one of the genes had a threshold (Ct) greater than 37 and the other less than 37. In the event of undetermined results, the corresponding RNAs were retested by performing a new RT-PCR. If the results were once again inconclusive, the participant was resampled and retested.

**Sample size:** Lorentz's formula was used to determine the minimal sample size. We used a margin of error of 5%, a 95% confidence level equivalent to Z =1.96, and a prevalence P of 0.5 as the prevalence of SARS-CoV-2 was unknown at the time of the study. A minimum sample size of 384 was determined.

**Quantitative variables:** we categorized our population into specific age groups based on their exposure risks and susceptibility to SARS-CoV-2 infection. The group “below 5 years” includes infants and young children. The “5-15 years” category encompasses school-age children, while “15-25 years” captures young adults who are more likely to attend social gatherings. The “25-45 years” group represents adults in their prime working years, and the “45-55” and “55-65 years” categories represent middle-aged adults who may have an increased risk of comorbidities. Lastly, the “≥ 65 years” age group includes the elderly, who often have fragile immunity due to age and are at a higher risk for comorbidities.

**Data analysis:** the Statistical Package for Social Sciences (IBM SPSS version 25 software) was used for statistical analysis. The chi-2 test was used to compare proportions and a value of p<0.05 was considered significant.

**Ethical consideration:** this study received Ethical approval from the national ethics committee (N° 2020/05/1224/CE/CNERSH/SP).

## Results

**Sociodemographic analysis:** from 16 March 2020 to 31 December 2022, a total of 142,850 participants from all regions of Cameroon were analysed. The majority were from the Centre region. The age of the participants ranged from 1 to 99 years, with a mean of 39.2±15.6 years. Participants aged between 35 and 45 years were the most common, and the most represented gender was male (Table 1). The M/F sex ratio was 1.32.

**Table 1:** characteristics of participants by year

	2020		2021		2022		TOTAL	
**Sex**	**N**	**%**	**N**	**%**	**N**	**%**	**N**	**%**
Female	19 172	41.3	30 845	44.1	11 289	42.8	61 306	43
Male	27 231	58.6	38 958	55,8	15 035	57.1	81 225	57
Sub-total	46 403	100	69 803	100	26 324	100	142 531	100
**Age group**								
<5	236	1.04	964	1.4	214	0.8	1 414	1.2
[5-15[	1 130	4.9	3 703	5.6	1114	4.3	5 947	5.2
[15-25[	2 658	11.7	6 412	9.7	2522	9.7	11 592	10.1
[25-35[	5 661	25.0	14 123	21.4	5 661	21.8	25 445	22.2
[35-45[	5 754	25.4	16 069	24.3	6 469	25.0	28 292	24.7
[45-55[	3 938	17.3	13 089	19.8	5 259	20.3	22 286	19.4
[55-65[	2 319	10.2	7 778	11.8	3 057	11.8	13 154	11.5
≥65	943	4.1	3 722	5.6	1 567	6.0	6 232	5.4
Sub -Total	22 639	100	65 860	100	25 863	100	114 362	100
**Region**								
Adamawa	3	0.006	0		0		3	0.002
Centre	43 320	96,5	68 733	99.8	26 270	99.8	138 323	98.8
East	66	0.1	0		0		66	0.04

**Figure 1:**
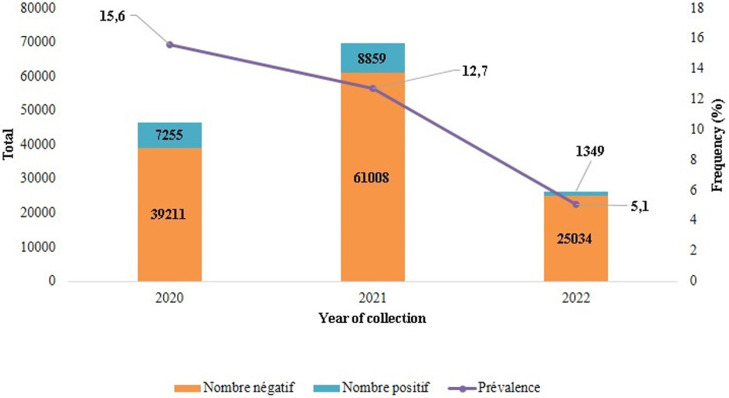
detection frequency of SARS-CoV-2 in Cameroon between 2020 and 2022

**Figure 2:**
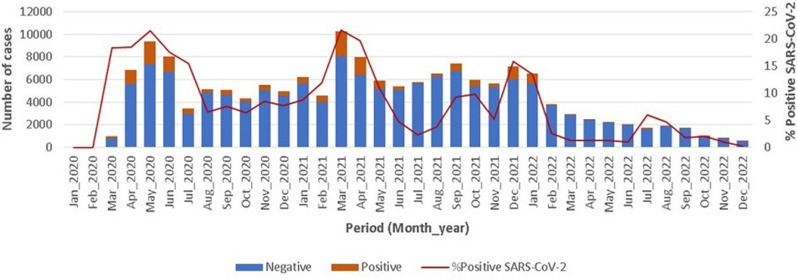
distribution of SARS-CoV-2 cases in Cameroon

**Main results:** from 2020 to 2022, the overall detection rate of SARS-CoV-2 was 12.2% (17,463/142,850). In 2020, the detection rate was 15.6% (7255/46,466), in 2021 12.7% (8859/69867) and in 2022 5.1% (1349/26,383) (Figure 1). The detection rates were higher in May 2020, March 2021, September 2021 and December 2021, corresponding to the four peaks of the COVID-19 epidemics between 2020 and 2022, as shown in Figure 2.

**Figure 3:**
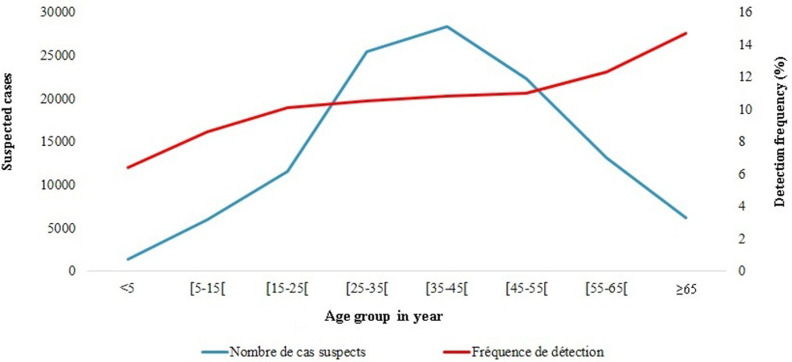
detection frequency of SARS-CoV-2 and suspected COVID-19 cases according to age

**Descriptive analysis:** we obtained a significantly higher SARS-CoV-2 positivity rate in 2020 compared to the subsequent years. The distribution of positivity rates by age showed that there were significantly higher SARS-CoV-2 detection rates in the age groups starting from 15 years and older, ranging from 8.7% in the 15-25 age group to 14.7% in persons aged over 65 years. With respect to region, significantly higher detection rates were found in the Littoral, East and South regions. We observed no difference in the prevalence of SARS-CoV-2 by gender (Table 2). The detection frequency was illustrated according to the age groups in Figure 3. The SARS-CoV-2 detection rate increases with age, in contrast to the number of suspected cases.

**Table 2:** SARS-CoV-2 positive results with respect to participant characteristics

	Total Tested N	SARS-CoV-2 Positive Results n (%)	p-Value	OR (IC 95%)
**Years**				
2022	26383	1349 (5,1)	Ref	1
2021	69867	8859 (12,7)	<0,001	2,69 (2,54-2,86)
2020	46466	7255 (15,6)	<0,001	3,43 (3,23-3,65)
**Age group (years)**				
<5	1414	90 (6,4)	Ref	1
[5-15]	5947	511 (8,6)	0,006	1,38 (1,10-1,74)
[15-25]	11592	1169 (10,1)	<0,001	1,65 (1,32-2,06)
[25-35]	25445	2672 (10,5)	<0,001	1,73 (1,39-2,14)
[35-45]	28292	3047 (10,8)	<0,001	1,78 (1,43-2,20)
[45-55]	22286	2445 (11,0)	<0,001	1,81 (1,46-2,25)
[55-65]	13154	1615 (12,3)	<0,001	2,06 (1,65-2,56)
≥65	6232	915 (14,7)	<0,001	2,53 (2,02-3,17)
**Sex**				
Female	61306	7676 (12,5)	0,004	1,04 (1,01-1,08)
Male	81224	9765 (12,0)	Ref	1
**Region**				
Centre	138201	16519 (12,0)	Ref	1
Adamawa	3	0 (0,0)	0,999	0,00
East	66	21 (31,8)	<0,001	3,44 (2,05-5,77)
Littoral	500	168 (33,6)	<0,001	3,73 (3,09-4,49)
North	4	0 (0,0)	0,999	0,00
North West	12	1 (8,3)	0,701	0,67 (0,09-5,19)
West	433	63 (14,5)	0,097	1,25 (0,96-1,64)
South	384	75 (19,5)	<0,001	1,79 (1,39-2,30)
South West	228	25 (11,0)	0,646	0,91 (0,60-1,37)

## Discussion

The worldwide reported COVID-19 pandemic also significantly affected Cameroon, causing several cases of infection. This study frequency of detection of SARS-CoV-2 from 142,850 nasopharyngeal swabs collected from all regions of Cameroon between March 2020 and December 2022 and analyzed at the Centre Pasteur Cameroon. The analysis showed a detection rate of 12.2% and the risk factors for infection were being over 65 years of age and being from the Littoral region. The study population consisted mainly of patients aged [35-45], with a predominance of male patients (Table 1). Our results are similar to those obtained in an epidemiological study in Cameroon, where predominant age group was 30-49 years [7]. Most of the samples received at the Centre Pasteur of Cameroon were from the Centre region, as this is where the main diagnostic centres for COVID-19 were located.

In our study, we observed a decreasing detection frequency over the years, from 15.6% in 2020 to 12.6% in 2021 to 5.1% in 2022 (Figure 1) that is a decrease of up to 11%. This significant decrease in the positivity rate could be the result of epidemic management in our context to limit the spread of SARS-CoV-2. To this end, the Cameroon government had implemented a number of prevention strategies and safety measures like containment, awareness campaigns, rapid diagnosis with the use of antigen rapid diagnostic tests (AgRDTs) or decentralization of molecular testing to cover all regions, and improved genomic surveillance aided by sequencing platforms from within and outside the country [8]. In 2021, Fokam *et al*reported a similar detection rate of 12.7%, which is consistent with our results [9]. Our results are also consistent with the detection rate (8.1%) obtained in an epidemiological study conducted in Douala, the economic capital of Cameroon and therefore a rather heterogeneous city representative of the general population [10]. In fact, this previous study observed a detection frequency of less than 10% in 2022, indicating a decrease in the rate of routinely detected SARS-CoV-2 positives, in contrast to the rate observed in 2020, at the beginning of the COVID-19 epidemic in Cameroon.

Four peaks of COVID-19 epidemics were observed during the study period, in May 2020, March 2021, September 2021 and December 2021 (Figure 2). The highest peak was observed in May 2020 and the lowest peak in September 2021. These peaks may reflect the emergence of the different identified SARS-CoV-2 variants, as experienced by several other countries. In another study from Cameroon, partial sequencing of the SARS-CoV-2 spike gene identified several variants between January and December 2021: alpha, beta, gamma, delta, mu, and omicron [11]. Of these variants, the last two peaks in Cameroon were reported to be driven by the delta and omicron variants, respectively. In addition, our results are consistent with those of Fokam *et al*. who studied the dynamics of SARS-CoV-2 spread in Cameroon between 2020 and 2022. They showed that the first wave in Cameroon occurred between week 18 and 33 in 2020, and that only the original lineage was responsible for this peak. In weeks 2 to 22 of 2021, they obtained the co-circulation of the alpha and beta variants, corresponding to wave 2, and the appearance of the delta variant, corresponding to wave 3, in weeks 36 to 46 of 2021. Between week 50 of 2021 and week 5 of 2022, they observed the circulation of only the omicron variant, corresponding to wave 4 [12]. In addition, the lower levels of subsequent peaks could be explained by the fact that the collective immunity acquired by the vaccination introduced in Cameroon in 2021 or by natural immunisation had a better effect on the transmission of SARS-CoV-2 [13].

With regard to age, SARS-CoV-2 cases increased with increasing age and the highest frequency of detection was observed in patients over 65 years of age (Figure 3), although they were among the least represented in our study population. A recent report by Monamele *et al*. on SARS-CoV-2 cases detected through the influenza surveillance system also found that all age groups above 15 years were associated with a higher frequency of SARS-CoV-2 infection [11]. The co-morbidities and reduced immune system functionality generally observed in the elderly would explain the higher detection frequency in this group of patients, predisposing them to a higher incidence of SARS-CoV-2 infection. A cohort study conducted in Yaounde on the clinical profile and factors associated with COVID-19 reported that in the age group 60-69 years, diabetes, HIV infection, lung disease, dyspnoea and fatigue predispose to more severe SARS-CoV-2 infection [14]. Similarly, a survey conducted in Cameroon on the prevalence of SARS-CoV-2 among adult populations in Yaounde and Douala showed that comorbidities were associated with seropositivity [15]. Our result is comparable to the detection frequency in patients over 70 years of age reported by Moguem Soubgui *et al*. They reported a more than seven-fold increased risk of SARS-CoV-2 infection in the age group over 70 years, with a positivity rate of 23.5% compared to 6.1% in patients aged 30-39 years.

A limitation of this study was the lack of some important data on participants characteristics. In fact, not all socio-demographic and clinical data were systematically recorded on the individual case identification forms that accompanied each sample sent to the laboratory. It was therefore not possible to describe more fully other potential risk factors that might have been associated with increased SARS-CoV-2 detection, such as travel history and vaccination.

## Conclusion

The overall SARS-CoV-2 positivity rate in Cameroon was 12.2% and decreased over the years, probably due to compliance with the Cameroon government´s barrier measures to reduce transmission. Although, the COVID-19 pandemic has been declared over [16], it is important to remain vigilant and to continue to test for SARS-CoV-2 in persons presenting with respiratory illness in order to remain on alert for future outbreaks of SARS-CoV-2.

### What is known about this topic

- Several data obtained from studies conducted by different authors around the world report detection frequency figures during the COVID-19 pandemic;
- In Cameroon, the frequency of detection of SARS-CoV-2 in the cities of Yaoundé and Douala was 12.7 in 2021 and 8.1 in 2022, respectively;
- The rate of COVID-19 positives detected between 2020 and 2022 decreased over time.

### What this study adds

- Our study included collection sites from several regions of Cameroon and was conducted over two years;
- It therefore reflects the epidemic situation during the different waves of the COVID-19 epidemic that we experienced between 2020 and 2022;
- The study updates data on the frequency of detection of SARS-CoV-2 in Cameroon to include the years 2020, 2021 and 2022.
